# The Effect of Adult Children's Education Attainment on Their Parents' Cognitive Health: An Intergenerational Support Perspective

**DOI:** 10.3389/fpubh.2022.744333

**Published:** 2022-02-09

**Authors:** Ying Xu, Yaping Luo

**Affiliations:** School of Public Policy and Management, Guangxi University, Nanning, China

**Keywords:** cognitive health, adult children, education attainment, intergenerational support, health

## Abstract

The purpose of this study is to examine the relationship between adult children's education attainment and their parents' cognitive health, and to further explore the mechanism of intergenerational support. Based on empirical analysis of data from China Health and Retirement Longitudinal Survey, our study finds a positive association between children's educational attainment and parents' cognitive health. This correlation is provided for by emotional communication of informal caregiving, financial support, and healthy behaviors shaped in the parents by adult children. The strength of the effect varied by the adult child's gender. While sons' education attainment significantly improves parental cognitive parameters through informal caregiving, financial support, and development of healthy behaviors, the effect of daughters' education derives from financial support and healthy behaviors, not being related to informal caregiving. The study enriches the evidence on the mobility of children's human capital toward their parents and provides practical insights for advancing children's participation in family caregiving.

## Introduction

Research on intergenerational mobility of human capital emphasizes the impact of parents on their children. Studies from both developed and developing countries have consistently confirmed that parents' education promotes human capital accumulation in children (1–4). However, the impact of adult children's education attainment on their parents' health has so far remained a blind spot in researches. The established literature has paid less attention to this correlation, unlike the parent-child direction of the human capital mobility, and the findings vary. On the one hand, studies in the United States, Germany, and other Western countries do not conclusively show that adult children's education predicts parents' health patterns (5–7). On the other hand, studies from developing countries such as Mexico and South Africa provide opposite evidence suggesting that adult children's education is beneficial to their parents' health (8, 9). The controversial difference in evidence has aroused widespread concern in the academic community. Several articles have explored the relationship between adult children's education and the health of Chinese parents, but mainly focused the assessment on depression, general health parameters, and parental survival status (10–12), with little attention paid to the effect of adult children's education on parents' cognition.

Recent research suggests that adult children's education attainment affects their parents' health by the means of increasing resources. Educational attainment with concomitant caregiving and economic resources of adult children produces a significant spillover effect on parent's health (13, 14), which exceeds other socioeconomic status factors (15). The benefits generated by adult children's education not only provide parents with the economic resources to maintain their health, but also play a role in caring for and managing their health by developing health awareness and storing health knowledge. Traditionally, parents invest in their children's education in exchange for help and health support in old age (16). Adult children's education translates into the health advantage for parents through intergenerational support, which includes financial assistance, care and spiritual comfort. Thus, intergenerational support may be the mechanism that connects adult children's education with parents' cognitive health.

Children's support is a major factor in the social support system for sick elderly individuals in developing countries (17). In regions such as Africa, Asia, and Latin America, elderly people usually live with at least one child or extended family member to facilitate children's support for living and health needs (18). At the same time, the stability of children's support has been impacted by rapid aging and advanced age. In developing countries in Asia, in particular, coupled with smaller, nuclear family size, parent-child separation and lack of care and emotional comfort from children have become major causes of cognitive decline, increased health risks, and even suicidal tendencies among older adults (19–22). These countries often lack social security resources and have weak social support mechanisms to address the health problems of older populations. Therefore, there is an urgent need to rediscover the value and function of family and provide policy support to promote informal caregiving and family supporting.

This study investigated the impact of adult children's educational attainment on parents' cognitive health, in China, as a developing country with a large population, to address the existing gap in literature. Further evidence was explored to identify how intergenerational support functions, which provides lessons for other countries to address similar concerns. Specifically, based on China Health and Retirement Longitudinal Survey (CHARLS) data (2011–2018), benchmarking regression to test the relationship between adult children's educational attainment and parents' cognitive health was conducted using the Ordinary Least Squares (OLS) method and the Ordered Probit (Oprobit) model. The empirical results indicate that the relationship between adult children's education attainment and parents' cognitive health is positively correlated. Multiple robustness tests by replacing the dependent variable, using new data from 2018, checking the parallel trend hypothesis that Oprobit holds, excluding genetic interference and testing the omitted variables confirm this finding. The mechanism this correlation relies on is intergenerational support provided by adult children to their parents, including informal caregiving, financial support, and influence on parental health behaviors. Intensity of the effect varies by gender of adult children, sons' education significantly enhanced parents' cognitive function through caregiving, financial support and healthy behaviors formation, while daughters' education was mainly about financial support and healthy behaviors, having no significant effect on informal caregiving.

This study makes three main contributions to the research in the effects of adult children's education on parental wellbeing. First, the research on the relationship between adult children's education attainment and parents' cognitive health and related literature was enriched in terms of gender differences. In contrast to previous studies, the study found that education attainment of adult daughters improved parental cognitive status primarily by influencing financial support and health behaviors, not having significant impact on informal caregiving. This finding informs future discussions of the differential impact of gender-diverse adult children on parental health. Second, this study introduces social activities in the cognitive assessment and discusses the relationship between social activities and cognition in the Chinese context. This approach not only expounds the potential mechanisms by which children's education attainment affects parents' cognitive health, but also provides empirical evidence from developing countries. Finally, a proven positive effect of adult children's education on parents' cognition may confound the effects of unobservable factors (e.g., genetic inheritance). In order to exclude the influence of genetic factors on the estimated results, the study identifies biological children by separating them from non-biological (including adopted or step children of the spouses in reconstituted families).

The body article is organized as follows: the second part reviews the relevant literature; the third part proposes the mechanism for intergeneration support studied based on the theoretical background; the fourth introduces the model, data, and key variables used in the study; the fifth presents the empirical analysis; the sixth analyzes the heterogeneity and the mechanism; and the last section summarizes and discusses this study's conclusions and implications.

## Literature Review

Declining cognitive levels and the serious consequences of this process have received extensive attention from the interdisciplinary teams in medicine, sociology and economics. From the medicine perspective, cognitive decline eventually deteriorates into Alzheimer's disease, the prevalence of which increases with age. With external help, “early identification, early intervention” strategy constitutes a critical means of delaying the onset of the disease (23). Sociology stresses the necessity of family support in preventing cognitive decline in older parents. Living arrangements, caregiving and intergenerational relationships are common factors that affect the psychological and mental health of elderly individuals (24, 25). Recent studies have addressed the impact of individuals' education level on cognitive health rather from a socioeconomic status perspective. Empirical evidence from America, the UK, Germany, and China indicates that educational experiences and their associated resources can buffer cognitive impairment in old age (26–29). The key to delaying cognitive decline in older adults, therefore, lies in adequate caregiving resources and socioeconomic resources.

Adult children's education attainment is considered an essential source of family resources for parents (10, 30), effectively meeting the financial, caregiving, and emotional demands of older parents. Previous research has explained the differential impact of education on parental health in the light of resource multiplication and resource substitution (31). On the one hand, resource multiplication presupposes that the educational achievements of adult children and the related benefits amplify the health advantages of parents. Specifically, as the primary health advantage group, they benefit more from the education of their children. Following this logic, our study argues that adult children's education attainment contributes to maintaining a good cognitive state of the parents and preventing a rapid decline in cognitive level. On the other hand, resource substitution is defined as the process in which resource-disadvantaged groups receive more educational benefits (32, 33). The concept suggests that the resources generated by adult children's education attainment can “compensate” the insufficiency or gaps in parental health resources. Parents who rely on their own single resource are more vulnerable to health risks, the resources provided to them by their children become essential. In summary, both theories essentially capture the idea that children's education increases the health resources of parents.

Resource multiplication and resource substitution reflect two different ways in which education impacts health, but both consistently indicate that adult children's educational resources protect or improve parental cognitive levels. To be specific, the education attainment of adult children shapes parents' cognitive health in three ways: health status, behavior, and psychology. Education increases the economic resources available for health maintenance, which in turn improves health status, reduces mortality, and extends life expectancy (34, 35). In terms of health behaviors, education builds health awareness, prompts healthy lifestyle habits, and reduces the inclination toward health risk behaviors such as smoking and alcohol abuse (28, 36). Psychologically, children gain a stable social-financial status through education, which reduces parents' negative emotions and psychological stress, thus reducing the likelihood of depression (37).

In East Asian cultural societies, the mechanism linking adult children's education attainment to their parents' cognitive health is intergenerational support. Recent empirical studies have pointed out that children's education brings economic and caregiving resources to improve parents' health, with little attention to health behaviors. Education of Korean adult children serves as cognitive health protection factor through transfer of economic resources to parents (38). China-based empirical studies suggest that adult children's education enhances parental health by providing financial resources and informal caregiving (11, 12). However, mechanism analysis from relevant studies in two countries did not demonstrate whether adult children's education plays a cognitive protective role by shaping their parents' health behaviors (such as avoiding smoking or attending social activities). Previous evidence on the effects of health behaviors on cognitive process, especially attending social activities, has been presented based on the studies conducted in developed countries, while our study provides empirical evidence from developing countries by focusing on Chinese parents, and therefore enriching research in the related fields.

The impact of adult children's education attainment on parents' cognitive health is complex. Although research has been conducted to demonstrate the positive effects of the education of adult Chinese children on parental cognition processes condition, two issues remain for discussion. One is the need to fully comprehend the mechanisms by which adult children's education shapes the cognitive health of parents. Another, issue still to be explored in depth is gender differences in intergenerational support behaviors.

## Theory and Mechanism

**Figure 1:**
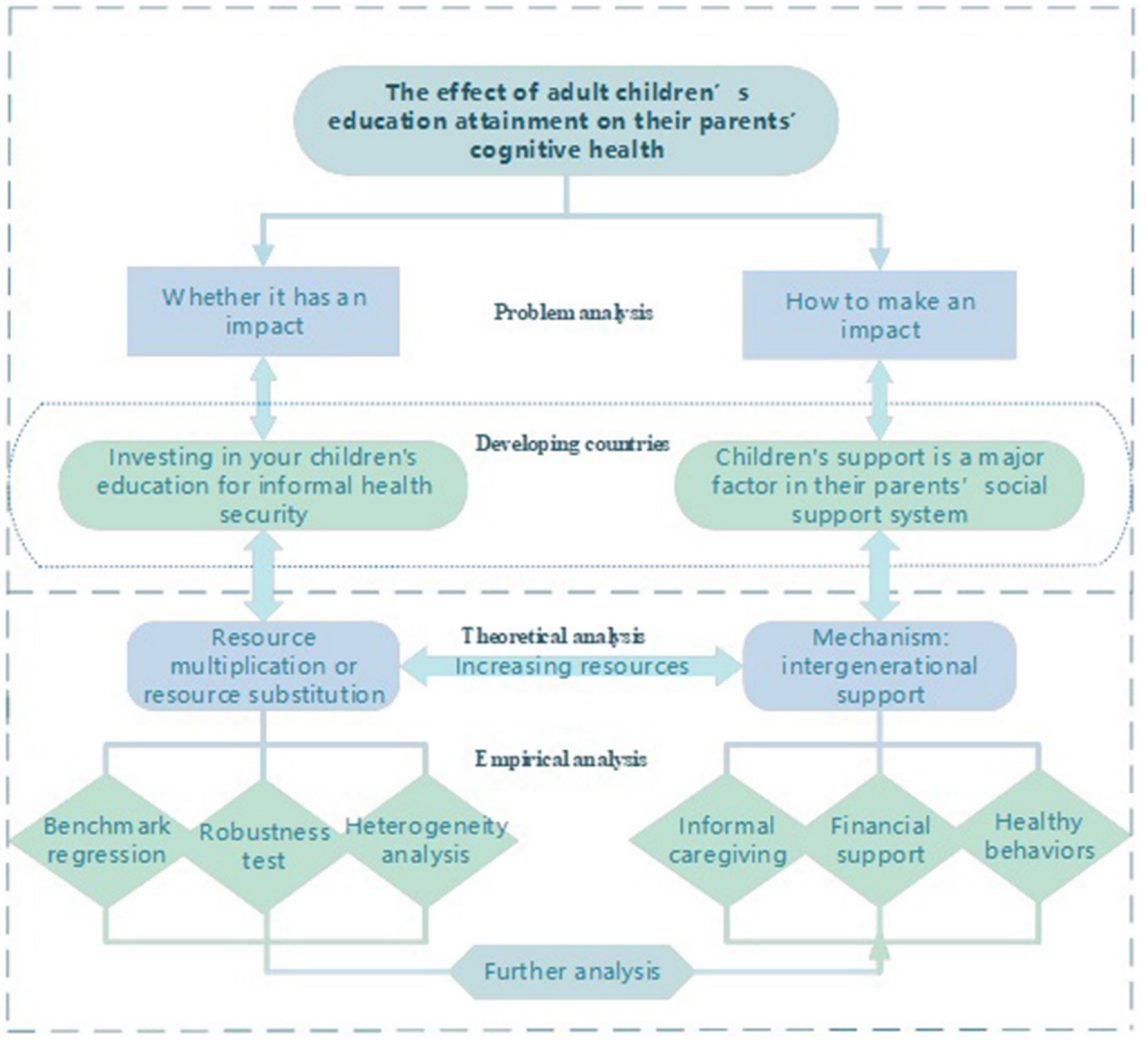
The analysis framework.

It has been documented that adult children's education attainment affects parental cognitive health by providing informal caregiving and financial resources. This provides a theoretical basis for our analysis of the channels between children's educational attainment and older parents' cognitive health. In addition, we have innovatively proposed a channel, i.e., shaping parents' health behaviors. The analysis framework of this paper is shown in Figure 1.

### Adult Children's Education Attainment and Possibility of Providing Caregiving

The family is an economic community unit. Families provide educational resources according to their children's abilities, while children return the benefits and help other family members and their parents in the future (39, 40). In terms of children's education and care for parents, more educated children are more likely to provide financial support for their parents, while less educated children tend to offer caregiving and emotional interaction. Although education brings considerable economic benefits to children, it also increases the opportunity cost of providing care. In general, the more educated groups are more migratory and more likely to move to areas with high levels of economic development (41). Caregiving limits the time available for work and evokes an increase in the cost of time, so children living far from their older parents would attempt to substitute caregiving absence with financial compensation (42). Given financial resource constraints and nurturing considerations, children with lower education will provide more sufficient care and emotional comfort than their more educated counterparts.

#### Mechanism 1

Informal caregiving is one way in which adult children's education attainment affects parents' cognitive health, but the effect of improving parents' cognitive condition through this route is less effective as children become more educated.

### Financial Rewards for Parents From Adult Children's Education Attainment

Education brings economic benefits to individuals and their parents. Education provides a pathway for people to advance their career and socioeconomical status (43, 44). The effect is particularly pronounced in China. With the urban-rural dual economic structure, education has become an important way for children to integrate into economically developed areas and obtain well-paid jobs in cities (45), thereby increasing their parents' economic resources and contributing to their socioeconomic status. More importantly, the phenomenon of sickness without medical care often occurs in China's elderly population because of low income (46). Financial support from children reduces financial barriers to health care access for older parents, whose health is promoted through increased use of health care services. Finally, the concept of “raising children for old age” has had a lasting impact on the practice of supporting for elderly in China. The financial support of children is an informal endowment resource, which to some extent relieves the mental burden of income insecurity in old age.

#### Mechanism 2

Educational attainment of adult children promotes parental cognitive health through increased economic resources.

### Adult Children's Education Attainment Shapes Parents' Healthy Behaviors

Regarding the relationship between children's education and parents' health behaviors, when children receive education, they normally acquire health knowledge and develop health awareness. This health knowledge is passed on to parents through their children to correct their unhealthy lifestyles, leading to a reduction in health risk behaviors such as smoking or alcohol and substance abuse, and setting the focus on daily exercise and active participation in social activities (47). Social activities serve as an effective way to relieve parental stress. Older parents experience a role change crisis as they exit the labor market. They may fail to adapt quickly to being idle at home, or being required to take responsibility for their grandchildren, which has a negative effect on their health (48, 49). Research has documented that active participation in social activities both alleviates the stress of role transitions in older parents, and serves as a way to maintain intellectual engagement (50–52).

#### Mechanism 3

Adult children's education attainment helps improve parental cognitive health by reducing parental smoking habits and promoting involvement in social activities.

## Methods and Materials

### Methods

The purpose of this study is to investigate the effect of adult children's education attainment on their parents' cognitive health, and construct econometric model as follows:


(1) $$\begin{array}{r} Mental\text{\_}status_{i}=\alpha_{0}+\alpha_{1}+Child\text{\_}edu_{j}+\alpha_{2}Controls_{ijf}+\mu \end{array}$$

where *i* refers to the father or mother, *j* is adult child, and *f* denotes the family household. Dependent variable is parental cognitive level, denoted as *Mental*_*status*_*i*_, measured using the memory for time orientation of respondents in the cognitive section of the CHARLS questionnaire. Adult children's education attainment is the core independent variable, noted as *Child*_*edu*_*j*_, α_1_ factor indicating that adult children's education attainment improves parents' cognitive level when α_1_ is positively significant. To ensure the accuracy of the estimation results, the article selected the demographic background of children and parents and the family background as control variables, presented as *Controls*_*f*_. The variable μ denotes the random error term. Benchmark regression is evaluated by the OLS method and the Oprobit model.

### Data and Sample

#### Data

The data used in this study is obtained from the China Health and Retirement Longitudinal Survey (CHARLS). This large-scale longitudinal survey project, hosted by Peking University, aims to provide the basic data for the study of aging in China. The CHARLS questionnaire is designed on the basis of the Health and Retirement Study (HRS) in the United States. Its sister databases ELSA (the Longitudinal Study of Adult Health) in the UK and SHARE (Survey of Health, Aging and Retirement in Europe), which means that the CHARLS questionnaire is scientifically sound. The baseline survey was conducted in 2011, covering 30 provinces or autonomous regions except for the Tibet Autonomous Region, Taiwan Province, and the Hong Kong and Macau Special Administrative Regions, involving 150 districts and counties nationwide. The survey targets individuals aged 45 and above, and mainly collects micro data on demographic backgrounds, health status and other aspects.

#### Sample

This study constructed a child-parent database using data from 3 waves of survey data collection from 2011 to 2015, from which the sample is selected to include families with adult children, with the respondent and his/her spouse as the core. The dependent variable was obtained from the cognitive component of the 2015's survey, other key variables were derived by tracking the two surveys in 2011 and 2013. To avoid reverse causality between education and cognition, the sample of minor children and the sample of adult children who were still receiving education during the survey period were excluded. It should be noted that, the average household in the 2015 survey had 3.9 children per household, and matching children to the heads of households makes the head's information approximately four times larger, but still ensures the validity of the data.

### Variables

Dependent variables. The dependent variables in this study are the constituents of the cognitive health level of the parents. Drawing from McArdle et al. (53), we used respondents' memory about time orientation to measure their cognitive health level, recorded as *Mental*_*status*. Given that measures of cognitive skills are multidimensional, a series of 100-7 calculations was used to assess the brain's processing of the task in robustness tests, called *Serial*_7*s*.

Core independent variable. The core independent variable is the highest educational attainment of the adult children, labeled as *Child*_*edu*. Considering a generally low and highly polarized national education level, the study directly uses the questionnaire's classification of the highest educational level of the children.

Control variables. With reference to previous studies, the article controls for common demographic characteristics. Parental dimension include age, gender, the Hukou status, residence, education, marital status, childhood health, basic health insurance, and life satisfaction. The child characteristics controlled for includes age, gender, occupation category, income level, child category, and the number of surviving children. The household level controls for total household income, housing size, running water, flush toilet, and interior neatness.

Channel variables. Channel variables are as follows: (1) Informal caregiving. The study used the frequency with which parents see and contact their children to measure the daily caregiving and emotional interaction that children provide for their parents. (2) Financial support. Measured by the total amount of in-kind and monetary support provided by the child in the past 12 months. (3) Health behavior. Parental health behavior was evaluated using smoking status and attendance of social activities. The main variables employed in the study are shown in Supplementary Table S1, and their descriptive statistics are drawn in the Table 1.

**Table 1:** Descriptive statistics.

	***N***	**Mean**	**S.D**.	**Min**	**Max**
**Dependent variables**					
Mental status	30,271	3.500	1.570	0	5
Serial-7s	30,244	2.610	2.000	0	5
**Core independent variable**					
Children's education	29,490	4.930	2.070	1	11
**Control variables**					
**Parents**					
Age	31,090	64.860	10.720	45	90
Gender	32,084	0.490	0.500	0	1
Hukou	27,291	0.170	0.380	0	1
Residence	31,949	0.160	0.370	0	1
Education	27,329	3.120	1.880	1	10
Marital status	32,077	2.180	1.770	1	7
Childhood health	29,674	2.740	1.150	1	5
Basic health insurance	31,854	0.800	0.400	0	1
Life satisfaction	29,871	2.630	0.800	1	5
**Household**					
Log of total income	21,460	8.370	2.010	0.690	15.420
Log of housing size	17,160	5.140	0.770	3.000	13.410
Flush toilet	24,571	0.400	0.490	0	1
Running water	31,610	0.740	0.440	0	1
Interior neatness	31,499	3.150	1.190	1	5
**Children**					
Children's age	31,553	37.550	10.100	18	63
Children's income level	22,504	5.610	2.250	1	12
Children's occupation category	24,489	4.320	1.470	1	6
Number of surviving children	31,905	3.950	2.050	1	16
Children category	31,413	1.340	0.620	0	4
**Channel variables**					
Average frequency of seeing (AFS)	30,287	5.120	2.090	1	10
Average frequency of contact (AFC)	26,630	4.800	2.350	1	10
Log of average financial support (LAFS)	26,303	6.930	1.320	3.28	13.3
Social activities (SA)	30,295	0.538	0.499	0	1
Ever smoked (ES)	31,912	0.460	0.500	0	1

*Note: Log refers to logarithm. Handling of outliers in this table: Tailor the age of parents and the age of children to between 0 and 99 years. Take the logarithm of the housing size and financial support and then tailor them to the range of 0–100*.

Table 1 reported the results of descriptive statistics for all variables required for the empirical analysis. The average education level of adult children is completed elementary school or junior high school level, and the overall education attainment is poor. Respondents had a higher mean score for *Mental*_*status* and a lower mean score for *Serial*_7*s*.

## Empirical Analysis

### Benchmark Regression

Table 2 reports the results of the OLS and Oprobit estimations of adult children's education attainment on parents' cognitive health. The empirical results indicate that the relationship between adult children's education attainment and parents' cognitive health is positively correlated. Specifically, adult children's education attainment is positively associated with parental cognitive status scores at the 1% significance level before any control variables are added. Given the effects of other variables on parental cognitive level, models (3) and (4) control for parental characteristics variables, child characteristics variables, and family-level variables. The results of model (3) indicate that for each higher tier of children's education, parents' cognitive scores will increase by 5.5%. Model (4) shows that, an increase in children's education is associated with a 0.056 Probit unit improvement in parents' cognitive status. Table 3 further provides its marginal effects. The results suggest that the probability of scoring 0–4 in cognitive status decreases by 0.3, 0.5, 0.6, 0.5, and 0.1 percentile points, respectively; the probability of scoring 5 increases by 2.1 percentile points.

**Table 2:** Results of benchmark regression.

**Dependent variable: Mental status**	**(1)**	**(2)**	**(3)**	**(4)**
	**OLS**	**Oprobit**	**OLS**	**Oprobit**
Children's education	0.245***	0.184***	0.055***	0.056***
	(0.00)	(0.00)	(0.01)	(0.01)
Parents' age			−0.022***	−0.019***
			(0.00)	(0.00)
Parents' gender			0.273***	0.217***
			(0.04)	(0.04)
Parents' Hukou			0.052	0.099*
			(0.05)	(0.06)
Parents' residence			0.195***	0.216***
			(0.06)	(0.07)
Parents' education			0.237***	0.201***
			(0.01)	(0.01)
Parents' marital status			−0.075***	−0.055***
			(0.01)	(0.01)
Childhood health			−0.050***	−0.049***
			(0.01)	(0.01)
Basic health insurance			0.021	0.002
			(0.05)	(0.04)
Life satisfaction			−0.060***	−0.060***
			(0.02)	(0.02)
Log of total income			0.016*	0.020**
			(0.01)	(0.01)
Log of housing size			−0.013	−0.013
			(0.02)	(0.02)
Toilet flushable			0.066*	0.054
			(0.04)	(0.03)
Running water			0.008	0.002
			(0.04)	(0.03)
Interior neatness			−0.031**	−0.031**
			(0.02)	(0.01)
Children's age			0.008**	0.008**
			(0.00)	(0.00)
Children's income level			0.027***	0.021***
			(0.01)	(0.01)
Children's occupation category			−0.014	−0.011
			(0.01)	(0.01)
Number of surviving children			−0.003	−0.003
			(0.01)	(0.01)
Constant term	2.322		3.896***	
	(0.02)		(0.24)	
*N*	28,019	28,019	5,371	5,371
*R* ^2^	0.110	0.036	0.220	0.079

Note: *, **, and *** mean significance at 0.1, 0.05, and 0.01 levels. Robust standard errors in parentheses. The last row in model 2 and model 4 is Pseudo_R^2^.

**Table 3:** The marginal effects of benchmark regression model (4).

**Mental status**	**0**	**1**	**2**	**3**	**4**	**5**
Children's education	−0.003^***^	−0.005^***^	−0.006^***^	−0.005^***^	−0.001^***^	0.021^***^
	(0.000)	(0.001)	(0.001)	(0.001)	(0.000)	(0.002)

Note: *** means significance at 0.01 levels. Robust standard errors in parentheses. Due to space limitations, we only show the marginal results for the core independent variables.

### Robustness Test

To ensure the robustness of the results of benchmark regression, the following strategies were used for testing: (1) replacing the dependent variable; (2) substituting the 2018 data for regression; (3) checking the parallel trend hypothesis that Oprobit holds; (4) using a biological children sample to exclude genetic interference; (5) testing the omitted variables.

#### Replace the Dependent Variable

In addition to time orientation, commonly used cognitive assessment dimension is calculated using a 100-7 series (*Serial*_7*s*). Supplementary Table S2 reports the effect of adult children's education attainment on parents' performance of the 100-7 task. Neither the OLS nor Oprobit regression results were significant before the inclusion of control variables. After the inclusion of control variables, the results of both model estimates show that children's education attainment is positively related to parental cognitive health. Specifically, at the 1% significance level, the OLS model estimates that one tier in adult children's education increases parental calculation scores by 5.3%. The marginal effects of Oprobit model in Supplementary Table S3 show that, the probability of scoring 0, 1, and 2 in the parental calculation decreases by 0.6, 0.7, and 0.1 percentile points, respectively; the probability of scoring 5 increases by 1.4 percentile points. The results showed no marked difference from the benchmark regression, further validating the positive correlation between adult children's educational attainment and parents' cognitive health.

#### Substitute New Data

Considering that participants' cognitive health changes with age, the article uses the cognitive levels reported in 2018 for the investigation. Keeping the control variables and regression model consistent with the benchmark regression, the results were re-estimated. The results of each model in Supplementary Table S4 show that educational attainment of adult children increases parental cognitive scores by 3.8% at the 1% significance level. Comparing the OLS estimates in Table 2 with those in Supplementary Table S4 reveals a modest decrease of 0.017 units in parents' cognitive level in 2018 vs. 2015. This measure demonstrates that the education attainment of adult children has a positive effect on moderating the rapid decline in parents' cognitive health.

#### Check Oprobit Parallel Trends

The validity of the Oprobit model relies on the assumption of parallel trends, which is expressed in the study as different coefficients for adult children's education attainment but parallel intercept terms. For this purpose, goprobit was used to perform a parallel trend test on the Oprobit model. As shown in Supplementary Table S5, the core independent variable corresponding to goprobit are statistically significant for all five coefficients of the dependent variable. This finding points to the robustness of the previous results.

#### Differentiation of Biological Children From the Total Sample

Educational attainment is associated with factors such as genetics and family characteristics, which can confound the estimates when these factors are unobservable. The children sample in CHARLS is mixed with the following categories: (1) Your and your current spouse's biological child, (2) Your biological child, but not your (current) spouse's, (3) The biological child of your (current) spouse, but not yours, (4) Your or your spouse's adopted or foster child. Where interesting information of the biological mother or father of children in categories (2), (3) and (4) cannot be controlled because they are not available from CHARLS. If those children are included in the regressions, the estimates may be influenced by unobservable biological or family background factors. Therefore, the samples of category (2), category (3) and category (4) are excluded, and the regression of the retained category (1) is performed. In addition, the children category is added to the model (4) of Table 2 as a new control variable. The models' estimates are shown in Supplementary Table S6. Specifically, model (1) and model (2) are regressed on a sample of biological children [categories (1)], and the Oprobit model is selected. The marginal results in Supplementary Table S7 show that, the education attainment of adult children is significantly and positively associated with the probability of scoring 5 on parental cognitive level. The results of models (3) and (4) are consistent with the results of the baseline model after adding the control variable children category.

#### Omitted Variable Test

Although a range of variables that may affect parents' cognitive health and children's education attainment are controlled for in the benchmark regression, variables such as regional differences in levels of economic development, social security policy (54), etc. may still be omitted. For this reason, this study draws on the potential omitted variables test strategy proposed by Oster (55). This strategy calculates a test statistic δ based on the coefficient changes of the core independent variable with and without control variables and the maximum available *R*^2^. This study assumes that the effect of the omitted variable on the dependent variable is as important as the independent variable (*Children's education*) in the study, the coefficient of the omitted variable (α^*^) is calculated when δ = 1. The results can be judged to be robust when the magnitude of α^*^ is not significantly different from that of the benchmark regression coefficients. The test result of α^*^ in our study is 0.0546, which is statistically significant and does not vary greatly from the model 3 in benchmark regression (α = 0.055). This result indicates that, the effect of adult children's education on parents' cognitive health is still robust even when the omitted variable is considered.

## Further Analysis

### Heterogeneity Analysis

Multigenerational living patterns facilitate financial support and emotional comfort for the elderly from their children, which has a positive impact on the quality of life, satisfaction and health of parents in their old age (56). However, the impact of population mobility and family planning policy has driven multi-generational living a rare practice. This situation has led to two opposing considerations. One view is that positive intergenerational relationships ensure adequate family support for older adults and that parent-child separation harms the physical and mental health of parents (57). At the same time, other scholars argue that parent-child separation allows them to avoid intergenerational conflict and the financial burden of children on their parents, and is beneficial to parental health (58, 59). In this connection, the first and second columns in Table 4 explore the effects of adult children's education attainment on parental cognitive health under different living arrangements. The estimation results show that living with children and living apart from children are positively associated with parents' cognitive health at the 5% and 1% significance levels, respectively. Separated children have more influence on parental cognitive health than children living together with them. The result is contrary to previous studies that concluded that living arrangements with children have a positive effect on parents' physical and mental health (60, 61). We will analyze the reasons for the finding in the following study.

**Table 4:** Heterogeneity analysis results.

**Dependent variable: Mental status**	**(1)**	**(2)**	**(3)**	**(4)**
	**Living with children**	**Living apart from children**	**Son**	**Daughter**
Children's education	0.050**	0.061***	0.056***	0.053***
	(0.02)	(0.01)	(0.01)	(0.01)
Control variables	Yes	Yes	Yes	Yes
*N*	1,496	3,666	2,932	2,418
*Pseudo*_*R*^2^	0.078	0.083	0.087	0.075

Note: ** and *** mean significance at 0.05, and 0.01 levels. Robust standard errors in parentheses. Heterogeneity was tested using the Oprobit model. Due to space limitations, the estimated parameters of control variables are not reported in this table, and readers can connect us for the detail results.

In East Asian cultural societies, gender differences prevail in intergenerational support. Intergenerational exchange under the Korean patriarchal system emphasizes the investment of parents in their sons, who are expected to give them support in the old age. Thus, the education of sons is a better predictor of parental cognitive trajectories than that of daughters (38). A China-based study concluded the opposite, with daughters' education significantly increasing father survival through informal caregiving (12). Even though the two countries share a similar family culture, gender and intergenerational support correlate differently there. This paper also focuses considers on gender heterogeneity. Table 4 shows that both sons' and daughters' education impact on parental cognitive health passed the 1% significance test. In terms of the magnitude of the coefficients, the effect of sons' education was slightly stronger than that of daughters, but the causes of the differences will be examined later.

### Mechanism Testing

Table 5 investigates the underlying mechanisms by which adult children's education attainment influences parental cognitive health in terms of informal caregiving, financial support, and shaping healthy parental behaviors.

**Table 5:** Mechanism analysis.

	**(1)**	**(2)**	**(3)**	**(4)**	**(5)**	**(6)**
	**AFS**	**Mental status**	**AFC**	**LAFS**	**SA**	**ES**
Children's education	0.014		−0.051***	0.071***	0.041***	−0.044***
	(0.01)		(0.01)	(0.01)	(0.01)	(0.01)
Children's education * AFS		0.002** (0.00)				
Control variables	Yes	Yes	Yes	Yes	Yes	Yes
*N*	5,135	5,135	4,555	4,634	5,371	5,371
*Pseudo*_*R*^2^	0.009	0.078	0.025 (*Adj*_*R*^2^)	0.130 (*R*^2^)	0.027	0.456

Note: *, **, and *** mean significance at 0.1, 0.05, and 0.01 levels. Robust standard errors in parentheses. Due to space limitations, the estimated parameters of control variables are not reported in this table, and readers can connect us for the detail results.

In regard to informal caregiving channels, the first column assesses the frequency of seeing to describe daily care, and the regression results indicate shows that the effect of the adult children's education on the frequency of meeting between parents and children is not significant. However, the interaction term (*Children's education* ^*^AFS) is positively associated with the probability of increased parental cognitive state scores at the 5% level of significance. This suggests that education attainment does increase the frequency of seeing. Model (3) characterizes emotional communication using contact frequency (by communication tools such as phone, message, WeChat, mail or email). The results show that the average frequency of contact will increase by 5.1% for each tier of children's education level. Informal caregiving channels demonstrate that the positive effect of adult children's education on parental cognitive health derives from emotional contact rather than daily caregiving.

This conclusion is consistent with reality of labor transfer and weakened intergenerational ties. For one, more educated children are more migratory, daily care is geographically and temporally inaccessible (62). This group maintains emotional contact with their parents primarily through the communication tools, which are effective in protecting the mental health of parents as studies have shown (63, 64). For another, with the weakening of intergenerational family ties, the phenomenon of parents and less educated children living together but with disharmonious intergenerational relationships, lack of emotional communication and daily care is common (65), which is not conducive to parents obtaining support resources from their children to maintain mental health. Therefore, daily caregiving had little effect on parental cognition, but emotional contact through communication tools significantly enhanced parental cognition health. This observation also explains why the heterogeneity test showed that cohabiting children have less impact on parents' cognitive level than children living separately. Mechanism 1 therefore was partially validated by the data analyzed.

In terms of financial support, as shown in model (4), the education of adult children attainment significantly increases the economic resources transferred to parents. At the 1% significance level, each higher level of adult children's education will result in a 7.1% worth financial return to parents. Referring to models (5) and (6) represent social activity and smoking status, respectively. The estimation results suggest that education of adult children attainment promotes parents' participation in social activities and significantly reduces parents' smoking behavior. This finding reveals that the education of adult children does improve parental cognitive status by increasing financial support and shaping healthy behaviors. Both mechanism 2 and mechanism 3 are hence verified.

Table 6 shows the mechanism tests for adult children's genders. The effect of sons' education, whether through informal caregiving, financial support or health behaviors, was effective at a 1% level of significance. The interaction term between sons' education level and the average frequency of seeing their parents suggests that the investment in sons' education pays back in the form of parental caregiving. Daughters' education influences parents' cognitive health primarily through increasing financial support and shaping healthy behaviors, independent of informal caregiving. This is consistent with Hu's (66) findings that Chinese daughters may also provide more financial support than sons. The interaction term between daughters' education and average seeing frequency also illustrates that education by itself does not promote increased caregiving resources. There is a practical basis for this conclusion: Chinese daughters commonly live with their husbands after marriage, hence the residential isolation of daughters from their parents which increases the difficulty of informal caregiving. As a consequence, daughters' concern for their parents is more often reflected in financial support.

**Table 6:** Mechanism analysis by gender.

	**(1)**	**(2)**	**(3)**	**(4)**	**(5)**
	**AFS**	**Mental status**	**LAFS**	**SA**	**ES**
Sons' education	0.029***		0.064***	0.041***	−0.046***
	(0.01)		(0.01)	(0.01)	(0.02)
Sons' education * AFS		0.004*** (0.00)			
Daughters' education	−0.007		0.084***	0.037***	−0.049**
Daughters' education * AFS	(0.01)	−0.001 (0.00)	(0.02)	(0.02)	(0.02)
*N*	3,159	3,159	2,816	3,279	3,279
*Pseudo*_*R*^2^	0.001	0.046	0.120 (*Adj*_*R*^2^)	0.038	0.040

Note: *, **, and *** mean significance at 0.1, 0.05, and 0.01 levels. The above tests omit the reporting control variables, and the control variables selected are the same as in the benchmark regression. Robust standard errors in parentheses. Due to space limitations, the estimated parameters of control variables are not reported in this table, and readers can connect us for the detail results.

## Conclusions and Discussion

Identification of controllable factors that affect cognitive function is essential to address cognitive aging. Putting the family in the focus, investigating the effect of adult children's educational attainment on parents' cognitive health, and uncovering the underlying mechanisms have attracted scholarly attention. Especially in the context of changing family structures and weakening intergenerational support, it is relevant to discuss parents' investment in children's education and children's return of care provision to their parents.

This study investigated whether and in which way adult children's education attainment affects parents' cognitive function, using the data of the China Health and Retirement Longitudinal Survey (CHARLS). Empirical evidence found that the relationship between adult children's education attainment and parents' cognitive health is positively correlated. This conclusion is supported by robustness tests such as replacing dependent variable, substituting new data, testing the Oprobit parallel trends, controlling for genetic factors, and using the Heckman selection model. According to the heterogeneity analysis, education of children living together with their parents had less impact on parents' cognitive function than that of children living separately. The education of both sons and daughters positively contributes to the cognitive health of parents. Mechanism analysis revealed that the effect of adult children's education on parental cognitive performance derived primarily from emotional interactions by informal caregiving, financial support, and healthy parental behaviors shaped, while the effect of daily caregiving in informal caregiving was not statistically significant. This mechanism varies by gender of the children. Sons' education promotes parental cognitive health through informal caregiving, financial support and healthy behaviors prompted. Daughters' education, on the other hand, works mostly through financial support and shaping healthy parenting behaviors, rather than informal caregiving pathways.

The conclusions point to policy insights to promote health in older populations in developing countries. Children's educational human capital has a health effect on parents. Thus, developing countries should continue to improve their education policies and promote the accessibility of high-level education. Second, in countries with weak social security systems, intergenerational support protects the health of older adults. Rediscovering the role of the family in society and encouraging adult children to take the initiative in providing support to their older family members can help reduce the burden of social care and achieve healthy aging. Ultimately, adult children's education improves parental cognitive function by shaping their healthy behaviors, which reflects the increasingly important role of social activities for health promotion in old age as developing countries progress socioeconomically. Therefore, it is necessary to provide recreational spaces and social activities for the elderly as a way to enhance their well-being (67).

In contrast to previous studies, this study did not confirm that adult daughters provided more informal caregiving. The literature on family caregiving and intergenerational relationships consistently found that daughters are closer to their parents and more willing to provide informal caregiving than sons (68–70). However, this study discovered that educated daughters improved their parents' cognitive health not through informal caregiving, but through financial support and modeling of healthy behaviors. In contrast, sons' education significantly promoted parental health via all the three pathways: by providing informal caregiving and financial support and by shaping parents' health behaviors. There are two explanations that account for these two opposite conclusions. One is that although family residence patterns have changed, the tradition of adult daughters living with their husbands is still dominant, and residential segregation is not conducive to daughters providing informal caregiving for their parents. The other reason resides in the reciprocal exchange characteristic of intergenerational relationships. Male offspring are the primary owners of family property and the principal caregivers of their parents, and their support behavior is driven by self-interest motives (71, 72). Instead, daughters are marginalized in the paternal property distribution (73), thus have little incentive to provide for them, and intergenerational support is unstable. This area needs more research to explore the gender variability of parental care designs.

This study's limitations are related to cognitive function testing scope and data grouping and processing procedure. Considering the lower educational level of the parents and the validity of cognitive testing, we used only two dimensions, the time orientation test and the 100-7 series calculation, to measure parental cognitive functioning, while word recall and delayed recall were not used, which may lead to bias in the assessment of parental cognitive function. Second, the design of this study is different from Ma (11), which replaces all children's education with the schooling years of the most educated adult children. This paper considers the full account of the differences in education attainment of each child, but this in turn results in duplication of parent-related information. In addition, due to missing data for the spouse cognitive module, this paper has not determined whether there is parental gender heterogeneity in the effect of adult children's education attainment on parental cognitive health. Finally, we acknowledge that despite efforts, we could only demonstrate a strong correlation between adult children's education attainment and parents' cognitive health. Future research could explore the causal relationship by look for exogenous policy shocks and constructing quasi-natural experiments (11, 12).

## Data Availability Statement

The original contributions presented in the study are included in the article/Supplementary Material, further inquiries can be directed to the corresponding author/s.

## Ethics Statement

The studies involving human participants were reviewed and approved by National School of Development at Peking University. The patients/participants provided their written informed consent to participate in this study. Written informed consent was obtained from the individual(s) for the publication of any potentially identifiable images or data included in this article.

## Author Contributions

YX: conceptualization, writing-review and editing. YL: data analysis, methodology, writing-original draft. Both authors contributed to the article and approved the submitted version.

## Funding

This research was funded by the Ministry of Education of the People's Republic of China. Research Project is Study on the incentive mechanism of public participation in rural water environment management (21XJA630007).

## Conflict of Interest

The authors declare that the research was conducted in the absence of any commercial or financial relationships that could be construed as a potential conflict of interest.

## Publisher's Note

All claims expressed in this article are solely those of the authors and do not necessarily represent those of their affiliated organizations, or those of the publisher, the editors and the reviewers. Any product that may be evaluated in this article, or claim that may be made by its manufacturer, is not guaranteed or endorsed by the publisher.
