# Supplementary material for: The Effect of Adult Children's Education Attainment on Their Parents' Cognitive Health: An Intergenerational Support Perspective
Source: Front Public Health. 2022 Feb 9;10:744333. doi: 10.3389/fpubh.2022.744333 (PMC8864153; doi:10.3389/fpubh.2022.744333)
Supplement: Supplementary file 1 [file Table_1.docx]

***Supplementary Material***

**Supplementary Tables**

| Table 1. Description of the variables employed in the study. | | | | | |
| --- | --- | --- | --- | --- | --- |
|  | **Descriptions** | | | | |
| ***Dependent variable***  *Mental status* | Memory about time orientation: Year; Season; Month; Date; Week. One correct answer is scored as one point, with a range of 0-5 points. | | | | |
| *Serial-7s* | Numeracy: The procedure is to subtract 7 from 100 and execute it 5 times, the correct calculation is counted as one point, ranging from 0 to 5 points. | | | | |
| ***Core independent variable***  Children’s education | No formal education (illiterate); Did not finish primary school; Sishu/home school; Elementary school; Middle school; High school; Vocational school; Two-/Three-Year College/Associate degree; Four-Year College/Bachelor’s degree; Master’s degree and Doctoral degree/Ph.D.; assign values of 1-11, respectively. | | | | |
| ***Control variables*** |  |  |  |  |  |
| ***Parents***  Age | Age of year >=45 | | | | |
| Gender | 1=Male; 0=Female | | | | |
| Hukou | The household registration (Hukou) system in China, which categorizes each person by hukou type and hukou registration place. There are two types of hukou: agricultural (rural) hukou and non-agricultural (urban) hukou. 1=Non-agricultural Hukou; 0=Agricultural Hukou | | | | |
| Residence | 1= Urban=1; 0=Rural | | | | |
| Education | No formal education (illiterate); Did not finish primary school; Sishu/ home school; Elementary school; Middle school; High school; Vocational school; Two-/Three-Year College/Associate degree; Four-Year College/Bachelor’s degree; Master’s degree and Doctoral degree/Ph.D.; assign values of 1-11, respectively. | | | | |
| Marital status | Married with spouse present=1; Married but not living with spouse temporarily for reasons such as work=2; Separated=3; Divorced=4; Widowed=5; Never married=6 | | | | |
| Childhood health | 1=Excellent; 2=Very Good; 3=Good; 4=Fair; 5=Poor | | | | |
| Basic health insurance | 1=Yes; 0=No | | | | |
| Life satisfaction  ***Household*** | 1=Completely satisfied; 2=Very satisfied; 3=Somewhat satisfied; 4=Not very satisfied; 5=Not at all satisfied. | | | | |
| Total household income | Add up the annual income of each household member and take the logarithm | | | | |
| Housing size | Take the logarithm of the family housing size | | | | |
| Toilet flushable | 1=Yes; 0=No | | | | |
| Running water | 1=Yes; 0=No | | | | |
| Interior neatness  ***Adult children*** | 1=Excellent; 2=Very clear=2; 3=Clear; 4=Fair; 5=Poor | | | | |
| Children’s age | Children’ s age of year >=18 | | | | |
| Children’s income level | 1=No income source; 2=Less than 2000 yuan; 3=2000-5000 yuan; 4=5000-10000 yuan; 5=10000-20000 yuan; 6=20000-30000 yuan; 7=30000-50000 yuan; 8=50000-100000 yuan; 9=100000-150000 yuan; 10=150000-200000 yuan; 11=200000-300000 yuan; 12=More than 300000 yuan | | | | |
| Children’s occupation category | 1=Managers in an enterprise or the persons in charge of general affairs in government agency/ organization of political party/ public service organization; 2=Professionals and technicians; 3=Clerks; 4=Workers in business and services; 5=Workers in Agriculture, forestry, animal husbandry and fishery; 6=Operating personnel of production/ transportation equipment; 7=Others | | | | |
| Number of surviving children | Number of children who survived the survey period | | | | |
| Children category  ***Channel Variables*** | 1=The biological child of you and your (current) spouse; 2=The biological child of you, but not of your (current) spouse; 3=The biological child of your (current) spouse, but not of yours; 4=The adopted or foster child of you or your spouse) | | | | |
| Average frequency of seeing (AFS) | How often do you contact with: 1= Almost every day; 2= 2-3 times a week; 3=Once a week; 4= Every two weeks; 5= Once a month; 6=Once every three months; 7= Once every six months; 8= Once a year; 9= Almost never; 10=Others. Take the average of frequency of seeing. | | | | |
| Average frequency of contact (AFC) | How often do you contact with (on phone/by message/ on WeChat/ by mail/ by email): 1= Almost every day; 2= 2-3 times a week; 3=Once a week; 4= Every two weeks; 5= Once a month; 6=Once every three months; 7= Once every six months; 8= Once a year; 9= Almost never; 10=Others. Take the average of frequency of contact. | | | | |
| Log of average financial support (LAFS) | Total in-kind and monetary support provided by the child in the past 12 months, where in-kind is converted to monetary amount, averaged and then logged | | | | |
| Social activities (SA) | Social activities attended in the last month. 1=Yes; 0=No | | | | |
| Ever smoked (ES) | 1=Yes; 0=No | | | | |

Note: Sishu refers to the teaching institution established by families, clans or teachers themselves in old China, and is a kind of private school.

Table 2. Replace the independent variable

|  | (1) | (2) | (3) | (4) |
| --- | --- | --- | --- | --- |
| Dependent variable  ***Serial-7s*** | **OLS** | **Oprobit** | **OLS** | **Oprobit** |
| Children’s education | 0.261 | 0.158 | 0.053*** | 0.034*** |
| Control variables | (0.01)  Yes | (0.00)  Yes | (0.01)  Yes | (0.01)  Yes |
| Constant term | 1.348 |  | 2.740*** |  |
|  | (0.03) |  | (0.35) |  |
| N | 27994 | 27994 | 5371 | 5371 |
| $R^{2}$ | 0.07 | 0.027  ($Pseudo\_R^{2}$) | 0.17 | 0.072  ($Pseudo\_R^{2}$) |

Note: *, **, and *** mean significance at 0.1, 0.05, and 0.01 levels. Robust standard errors in parentheses. Due to space limitations, the estimated parameters of control variables are not reported in Table 2, and readers can connect us for the detail results.

Table 3. The marginal effects of ***Serial-7s***

| ***Serial-7s*** | 0 | 1 | 2 | 3 | 4 | 5 |
| --- | --- | --- | --- | --- | --- | --- |
| Scores |  |  |  |  |  |  |
| Children’s education | -0.006*** | -0.007*** | -0.001*** | -0.000 | 0.000*** | 0.014*** |
|  | (0.001) | (0.001) | (0.000) | (0.000) | (0.000) | (0.003) |

Note: *, **, and *** mean significance at 0.1, 0.05, and 0.01 levels. Robust standard errors in parentheses. Due to space limitations, we only show the marginal results for the core independent variables.

Table 4. Substitute new data (2018)

|  | (1) | (2) | (3) | (4) |
| --- | --- | --- | --- | --- |
| Dependent variable  ***Mental status*** | **OLS** | **Oprobit** | **OLS** | **Oprobit** |
| Children’s education | 0.226*** | 0.184*** | 0.038*** | 0.038*** |
|  | (0.00) | (0.00) | (0.01) | (0.01) |
| Control variables | Yes | Yes | Yes | Yes |
| Constant term | 2.341 |  | 3.947*** |  |
|  | (0.02) |  | (0.22) |  |
| N | 30408 | 30408 | 7894 | 7894 |
| $R^{2}$ | 0.110 | 0.037  ($Pseudo\_R^{2}$) | 0.230 | 0.079  ($Pseudo\_R^{2}$) |

Note: *, **, and *** mean significance at 0.1, 0.05, and 0.01 levels. Robust standard errors in parentheses. Due to space limitations, the estimated parameters of control variables are not reported in Table 4, and readers can connect us for the detail results.

Table 5. Check Oprobit parallel trends

|  | (1) | (2) |
| --- | --- | --- |
|  | ***Mental status*** | ***Serial-7s*** |
| Children’s education 1 | 0.098*** | 0.055*** |
|  | (0.024) | (0.015) |
| Children’s education 2 | 0.049*** | 0.045*** |
|  | (0.017) | (0.012) |
| Children’s education 3 | 0.052*** | 0.027*** |
|  | (0.013) | (0.011) |
| Children’s education 4 | 0.063*** | 0.036*** |
|  | (0.012) | (0.011) |
| Children’s education 5 | 0.054*** | 0.024*** |
|  | (0.010) | (0.011) |
| Control variables | Yes | Yes |

Note: *, **, and *** mean significance at 0.1, 0.05, and 0.01 levels. Robust standard errors in parentheses. Child's education level 1 to child's education level 5 represents the results of the goprobit test for each coefficient corresponding to the dependent variable after considering parallel trends.

Table 6. Robustness tests for differentiating between children category

|  | (1) | (2) |  | (3) | (4) |
| --- | --- | --- | --- | --- | --- |
|  | Biological children | |  | Add control variables | |
|  | ***Mental status*** | ***Serial-7s*** |  | ***Mental status*** | ***Serial-7s*** |
| Children’s education | 0.055*** | 0.034*** |  | 0.056*** | 0.032*** |
|  | (0.01) | (0.01) |  | (0.01) | (0.01) |
| Control variables | Yes | Yes |  | Yes | Yes |
|  |  |  |  | -0.056** | -0.030 |
| Children category |  |  |  | (0.03) | (0.03) |
| N | 3982 | 3982 |  | 5435 | 5435 |
| $Pseudo\_R^{2}$ | 0.0794 | 0.0746 |  | 0.081 | 0.071 |

Note: *, **, and *** mean significance at 0.1, 0.05, and 0.01 levels. Robust standard errors in parentheses. Due to space limitations, the estimated parameters of control variables are not reported in Table 6, and readers can connect us for the detail results.

Table 7. The marginal effects of Table 6

|  | (1) | (2) |  | (3) | (4) |
| --- | --- | --- | --- | --- | --- |
|  | Biological children | |  | Add a control variable | |
| Scores | ***Mental status*** | ***Serial-7s*** |  | ***Mental status*** | ***Serial-7s*** |
| 0 | -0.003*** | -0.006*** |  | -0.003*** | -0.006*** |
|  | (0.001) | (0.001) |  | (0.000) | (0.001) |
| 1 | -0.005*** | -0.008*** |  | -0.005*** | -0.007*** |
|  | (0.001) | (0.002) |  | (0.001) | (0.001) |
| 2 | -0.006*** | -0.001*** |  | -0.005*** | -0.001*** |
|  | (0.001) | (0.000) |  | (0.001) | (0.000) |
| 3 | -0.006*** | -0.000*** |  | -0.005*** | -0.000 |
|  | (0.001) | (0.000) |  | (0.001) | (0.000) |
| 4 | -0.002*** | 0.000*** |  | -0.001*** | 0.000*** |
|  | (0.000) | (0.000) |  | (0.000) | (0.000) |
| 5 | 0.022*** | 0.016*** |  | 0.021*** | 0.014*** |
|  | (0.003) | (0.003) |  | (0.002) | (0.003) |

Note: *, **, and *** mean significance at 0.1, 0.05, and 0.01 levels. Robust standard errors in parentheses. Due to space limitations, we only show the marginal results for the core independent variables.
